# Seascape and life-history traits do not predict self-recruitment in a coral reef fish

**DOI:** 10.1098/rsbl.2016.0309

**Published:** 2016-08

**Authors:** Marcela Herrera, Gerrit B. Nanninga, Serge Planes, Geoffrey P. Jones, Simon R. Thorrold, Pablo Saenz-Agudelo, Glenn R. Almany, Michael L. Berumen

**Affiliations:** 1 Red Sea Research Center, Division of Biological and Environmental Science and Engineering, King Abdullah University of Science and Technology, Thuwal, 23955-6900, Saudi Arabia; 2 USR 3278 CNRS EPHE, Centre de Recherches Insulaires et Observatoire de l'Environnement (CRIOBE), BP1013 Papetoai, Moorea, French Polynesia; 3 ARC Centre of Excellence for Coral Reef Studies, James Cook University, 4811 Townsville, Queensland, Australia; 4 Biology Department, Woods Hole Oceanographic Institution, Woods Hole, MA 02543, USA; 5 Instituto de Ciencias Ambientales y Evolutivas, Universidad Austral de Chile, 5090000 Valdivia, Chile

**Keywords:** larval dispersal, connectivity, parentage, sibship, Kimbe Bay, metapopulation

## Abstract

The persistence and resilience of many coral reef species are dependent on rates of connectivity among sub-populations. However, despite increasing research efforts, the spatial scale of larval dispersal remains unpredictable for most marine metapopulations. Here, we assess patterns of larval dispersal in the angelfish *Centropyge bicolor* in Kimbe Bay, Papua New Guinea, using parentage and sibling reconstruction analyses based on 23 microsatellite DNA loci. We found that, contrary to previous findings in this system, self-recruitment (SR) was virtually absent at both the reef (0.4–0.5% at 0.15 km^2^) and the lagoon scale (0.6–0.8% at approx. 700 km^2^). While approximately 25% of the collected juveniles were identified as potential siblings, the majority of sibling pairs were sampled from separate reefs. Integrating our findings with earlier research from the same system suggests that geographical setting and life-history traits alone are not suitable predictors of SR and that high levels of localized recruitment are not universal in coral reef fishes.

## 1. Introduction

Connectivity in marine metapopulations is predominantly driven by the exchange of pelagic larvae among relatively sedentary adult populations. Recently, the relative importance of dispersal versus local retention of larvae has received considerable attention (e.g. [1,2]), owing to the importance of these processes for gene flow, local demographics and the spatial management of fisheries [3].

**Figure 1.**
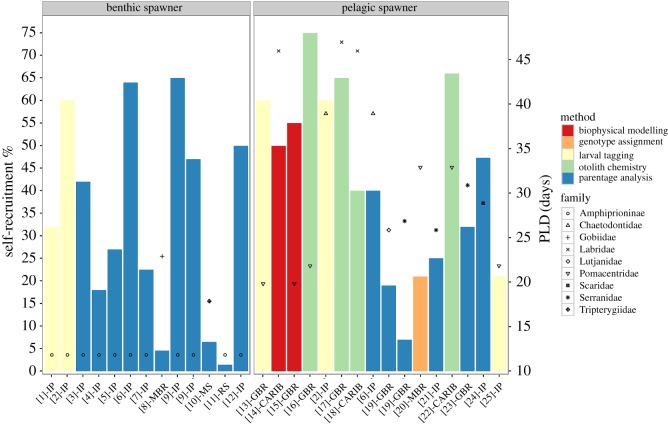
Self-recruitment (SR) estimates in coral reef fishes with different spawning modes (shown are the highest SR values (%) reported in each publication). Bar colours indicate the method used; symbols show the average pelagic larval duration (PLD) for each corresponding family. Labels on the *x*-axis correspond to the source (according to the reference list in the electronic supplementary material) and study regions: IP, Indo-Pacific; MBR, Mesoamerican Barrier Reef; MS, Mediterranean Sea; RS, Red Sea; GBR, Great Barrier Reef and CARIB, Caribbean.

To date, numerous studies have produced estimates of self-recruitment (SR; the proportion of all sampled recruits at a given location that had been locally produced) from different systems in a variety of coral reef fishes (figure 1). These studies have shown that levels of SR can be highly variable temporally both within a species [4,5] and among closely related species with similar life-history traits. Within anemonefishes of the genus *Amphiprion* alone, estimates of SR range from 0% [6] to 65% [7]. On the other hand, SR rates may also be remarkably consistent over time within species [8,9] and even among species with very different life-history characteristics [10].

The reef systems around Kimbe Island in Kimbe Bay, Papua New Guinea, have thus far produced remarkably consistent estimates of high SR for coral reef fishes. In this system, two species, albeit with different dispersal potential (*Amphiprion percula:* benthic brooder, pelagic larval duration (PLD) ≈12 days, high-site fidelity; butterflyfish *Chaetodon vagabundus*: pelagic spawner, PLD ≈38 days, large home range), were shown to exhibit high, consistent and remarkably similar SR levels over time [8,10,11], warranting the question whether Kimbe Island might be a hotspot for localized recruitment.

Here, we investigated patterns of local dispersal in Kimbe Bay in a third species of coral reef fish, the bicolor angelfish *Centropyge bicolor* (Pomacentridae), with similar life-history characteristics as *C. vagabundus* (pelagic spawner, PLD = 29–34 days [12]). We thus aimed to investigate the relative importance of seascape (defined here as the geographical setting of the habitat matrix) and specific life-history traits as determinants of SR in coral reef fishes.

## 2. Material and methods

Tissue samples of adult and recently settled *C. bicolor* were collected in April 2013 from nine different reef sites in Kimbe Bay (electronic supplementary material, figure S1 and table S1). In total, 255 potential parents and 426 juveniles were sampled. All 681 individuals were genotyped at 23 variable microsatellite loci (following [13]). We performed two types of kinship analyses: parentage and sibship. A maximum-likelihood approach was used to determine parent–offspring assignments as implemented in the software platform FaMoz [14]. Sibling groups within the juvenile sample pool were identified using Colony [15]. Full sibship was accepted upon a posterior probability exceeding 0.75. If localized recruitment was common in our study system, we would expect to find high numbers of siblings within short distances of each other (recruiting together) and the opposite if dispersal rates were high [16]. A *χ*^2^ test was implemented to assess differences in the proportions of sibling pairs and the entire juvenile sample at different distance classes.

Spatial autocorrelation performed in Genalex [17] was used to test the hypothesis of a random spatial distribution of the sampled juveniles by assessing the pairwise genetic similarity of individuals at different geographical distance classes. Samples were binned into 5 km distance class sizes, roughly resembling the real distances between islands. We ran 10 000 permutations to determine the 95% confidence intervals (CIs) around the null hypothesis of no spatial autocorrelation and 1000 bootstraps to estimate the 95% CIs of the autocorrelation index *r* for each distance class. Detailed descriptions of the methods are provided in the electronic supplementary material.

## 3. Results

Both Colony and FaMoz analyses yielded only two parent–offspring assignments (both single parents). One juvenile collected at Tuare Island was assigned to a parent sampled from the same location. At the reef scale of Tuare Island (*ca* 0.15 km^2^), this is equivalent to approximately 0.4–0.5% SR (considering that we had sampled 60–80% of the adult population). The other assigned juvenile travelled approximately 10 km northwest from a parent on South Bay Reef to also settle at Tuare Island. At the lagoon scale of all our sampling sites (approx. 700 km^2^), this equates to 0.5–0.8% SR (or 0.06% total if we consider SR at each reef individually).

**Figure 2.**
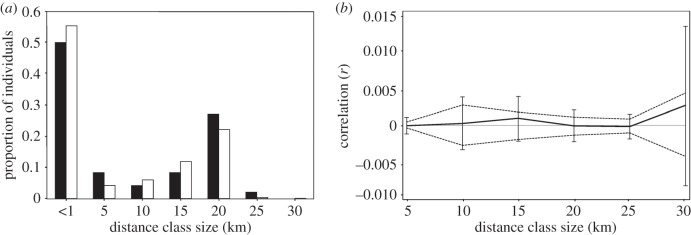
(*a*) Proportion of sibling pairs (black bars) and the entire juvenile sample (white) at 5 km distance classes. (*b*) Correlogram of the autocorrelation index *r* (black line) as a function of geographical distance for 426 juveniles in Kimbe Bay. The dashed lines represent the 95% CIs of the null hypothesis of a random distribution. Error bars represent the 95% CI determined by bootstrapping.

Sibship analysis performed in Colony confidently identified three pairs as full siblings; a further 42 pairs and one triplet had similar likelihoods of being full or half siblings. Of these potential sibling pairs, 53% recruited to separate reefs. The proportions of sibling pairs across distance classes closely matched that of all sampled juveniles (*p* = 0.99; figure 2*a*). Autocorrelation coefficients for all distance classes were close to zero and non-significant (figure 2*b*), suggesting that the genotype distribution of juveniles was spatially random across the study system.

## 4. Discussion

In contrast to virtually all previous studies on larval dispersal in Kimbe Bay, reporting consistently high levels of SR in different species of coral reef fishes [8,10,11], we found a near lack of SR and no apparent spatial structure in recruitment. Our results indicate that SR and local scale genetic connectivity patterns in coral reef fishes cannot readily be predicted from the local seascape or dispersal potential, commonly estimated from life-history traits such as reproductive mode and PLD. Instead, our findings suggest that other biological characteristics may deserve more attention, including larval and adult behaviour and mating and settlement strategies [18]. Local currents may be highly variable around coral reefs, leading to large temporal variability in settlement patterns of reef fishes [4,5,19]. While sampling did take place during the same season (spring) as in previous studies conducted in the region, we cannot rule out that annual variability in local oceanography may have led to differential settlement patterns in this study. A direct comparison of genetic data collected simultaneously on an anemonefish, *A. percula*, in the same reef system will give interesting insights in this regard (data not available yet). Either way, our findings highlight the unpredictable nature of connectivity and the need for further temporally replicated inter-species comparisons.

Recent research has focused mostly on seascape as a predictor of SR in coral reef fishes [9,20,21]. In theory, more isolated habitats would receive relatively higher levels of SR because of a lack of recruits from external sources [22]. This study, however, adds to the emerging notion that high levels of SR are not a universal phenomenon and that, regardless of the spatial setting of the habitat matrix, it cannot be assumed that populations will be able to be sustained by local production alone [7,16,20]. Consistent with the lack of local recruitment, *C. bicolor* seems to exhibit high levels of gene flow across the study area with no significant genetic structure among sites (electronic supplementary material, figure S2) and a seemingly random spatial distribution of recruitment (figure 2*b*).

The proportion of sibling pairs found in the juvenile sample was unusually high (approx. 25%). While we cannot make direct inferences about the origin of these individuals, the high proportion suggests that relatively few parents are responsible for successful juvenile recruitment. While the spatial distribution of sibling pairs seems to indicate local scale recruitment patterns (figure 2*a*) [16], it is important to note that more than 43% of these sibling pairs had equal probabilities of being half-siblings only. Moreover, the distribution of the proportion of sibling pairs at different distance classes closely resembled that of the entire juvenile sample, suggesting that the observed patterns simply reflected the spatial arrangement of juveniles in the study area. Overall, we urge caution when interpreting spatial sibling distribution in terms of dispersal scales.

By showing diametrically different patterns of local recruitment in a seemingly high SR system, we show that similar life-history traits (PLD and spawning mode) and/or the spatial structure of the habitat matrix cannot be assumed to serve as predictors for levels of SR in coral reef fishes. Understanding the physical and biological mechanisms underlying differences in SR is critical for the conservation of marine biodiversity through the design of networks of marine reserves. We therefore urge caution when using life history and/or seascape as predictors for SR in management decisions.

## Supplementary Material

Electronic Supplementary Material

## Data accessibility

Genotypic raw data is available at the Dryad Digital Repository: http://dx.doi.org/10.5061/dryad.f5r0p [23].
