# Supplementary material for: Seascape and life-history traits do not predict self-recruitment in a coral reef fish
Source: Biol Lett. 2016 Aug;12(8):20160309. doi: 10.1098/rsbl.2016.0309 (PMC5014023; doi:10.1098/rsbl.2016.0309)
Supplement: Electronic Supplementary Material [file rsbl20160309supp1.doc]

**Electronic Supplementary Material**

**Supplemental Methods**

Several sites with substantial coral reef habitat were targeted within the western half of the bay (figure S1). Specimens were captured by divers using hand nets, measured (total length – T_L_, cm), fin-clipped and released back into their original habitat. Fish that were too small to be fin-clipped (less than ~2 cm) were collected whole. The conspicuous fin clip ensured that the same fish were not resampled during the sampling period (10 days). Individuals were classified as juveniles (T_L_ ≤ 5 cm) or adults (T_L_ > 5 cm) on the basis of size at first sexual maturity [26]. Sampling occurred during the peak period of recruitment for many damselfish species in Kimbe Bay, April - May [27], coinciding with previous studies on settlement patterns of an ecologically similar species, *Chaetodon vagabundus* [6]. Variation in the number of individuals collected per site (table S1) was due to differences in population size, rather than sampling effort (e.g. even after extensive searches, no recruits were found in Kimbe Bommie).


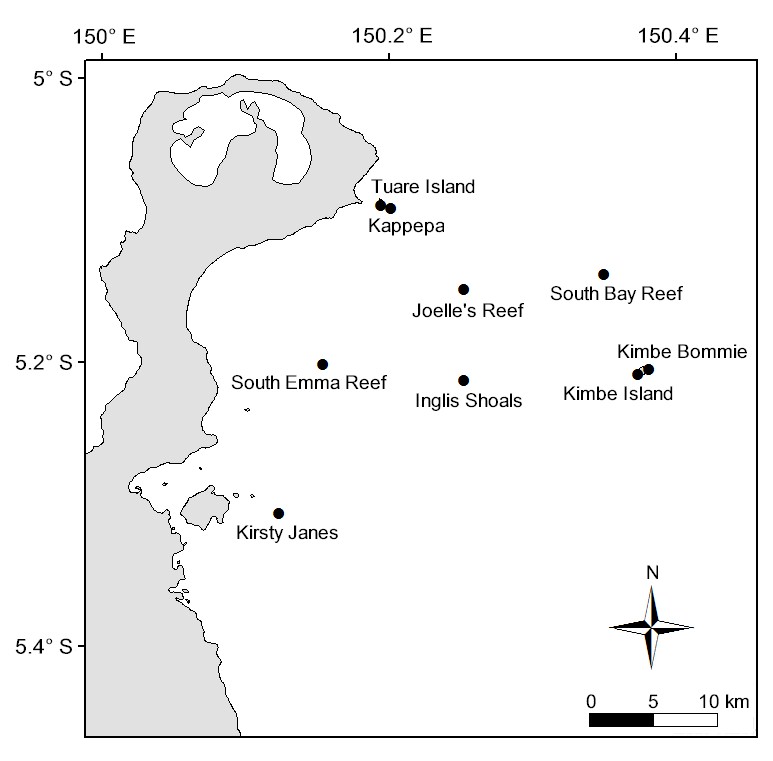


Fig S1. Map of Kimbe Bay in Papua New Guinea showing the sampling locations.

| Table S1. Sampling regime with original site names, GPS coordinates and number of samples collected per site. | | | | |
| --- | --- | --- | --- | --- |
| **Sampling location** | **Latitude** | **Longitude** | **Juveniles** | **Adults** |
| Inglis shoals | 5°12'43.16''S | 150°15'10.08''E | 3 | 8 |
| Joelle’s reef | 5°08'53.91''S | 150°15'07.30''E | 6 | 9 |
| Kirsty Janes | 5°18'24.19''S | 150°07'28.99''E | 2 | 2 |
| South Emma Reef | 5°12'05.87"S | 150°09'16.49"E | 18 | 14 |
| Kappepa | 5°05'30.37"S | 150°12'04.46"E | 2 | 1 |
| Tuare Island | 5°05'23.42"S | 150°11'41.02"E | 319 | 142 |
| South Bay Reef | 5°08'15.66"S | 150°20'57.87"E | 32 | 25 |
| Kimbe Island | 5°12'24.19"S | 150°22'21.65"E | 64 | 51 |
| Kimbe Bommie | 5°12'16.63"S | 150°22'46.88"E | 1 | 11 |

Deviations from Hardy-Weinberg Equilibrium (HWE) and linkage disequilibrium (LD) were evaluated using GENEPOP [28]. Loci that did not satisfied HWE assumptions after corrections for false discovery rates [29], as well as pairs of loci in LD were excluded from all subsequent analysis. We determined scoring error by re-analyzing one random plate (96 samples), re-running PCRs and genotype scoring.

We performed two types of kinship analyses: parentage and sibship. Parent-offspring assignments were determined using the software platforms COLONY [30] and FAMOZ [31]. In COLONY, genotyping error was set to 3.15% for all loci, according to our maximum empirically determined error rate. In FAMOZ, LOD scores were determined by the intercept of the frequency of distributions of 10 000 simulated parent-offspring pairs based on observed allele frequencies and another 10 000 pairs from the putative parental genotypes. Minimum threshold LOD scores for single parent- and parent-pair-offspring assignments were 7 and 23 respectively. We allowed a maximum of two mismatches. When calculating LOD scores, an error rate of 0.1% was taken into account. Simulations indicated a 99.5% likelihood of correct parent-offspring assignments.

Genetic structure within the samples was assessed using a Bayesian multi-locus clustering method implemented in STRUCTURE [32]. The algorithm assigns all individuals in the total sample to genetically distinct clusters (K) that are estimated using a log-likelihood approach of a pre-defined K value to minimize HWE and LD. An admixture model with sampling location as a prior was implemented. Allele frequencies were assumed to be correlated among populations. For each possible cluster (K = 1 to K = 10) simulations were carried out 10 times using 1 000 000 MCMC steps and a burn-in length of 50 000 iterations. Output files were later visualized and processed in STRUCTURE HARVESTER [33] and CLUMPAK [34] to assess the number of clusters K that best described the data according to the highest average maximum log-likelihood score. Finally, an analysis of molecular variance (AMOVA) was conducted in GENALEX [35] to examine population genetic differentiation. Global F_ST_ was tested for significance with 9 999 permutations.

**Supplemental Results**

No population structure was found in our sample across the study area (figure S2). STRUCTURE analysis identified K = 1 to be the most probable number of genetic clusters when combining all samples. The same result was observed when adults and juveniles were analyzed separately (data not shown). Additionally, F_ST_ estimates between individuals collected in Kimbe Island and the other sampling locations were low (global F_ST_ = 0.000) and not significantly different from zero (*p* = 0.714).


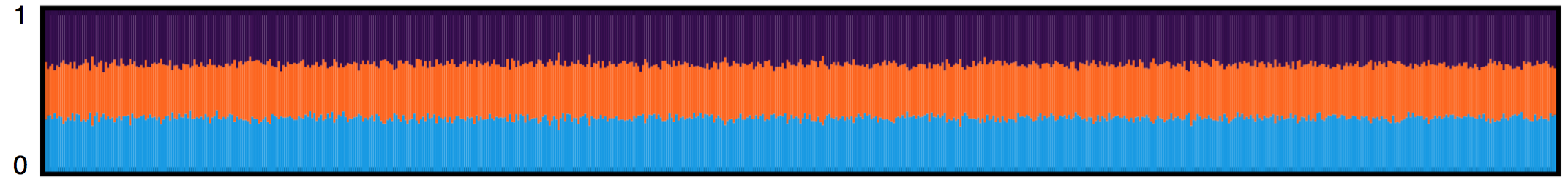


Fig S2. **Averaged bar plot graph of STRUCTURE results for K = 3 (K chosen for illustration purposes).** Each vertical line of the x-axis represents a single individual. Each color represents one of the 3 inferred clusters. Individual lines are broken into color segments, with lengths proportional to the probability of each inferred cluster assignment.

**Supplemental References**

1. Jones GP, Planes S, Thorrold SR. 2005. Coral reef fish larvae settle close to home. *Curr. Biol.* **15**, 1314–1318. (doi:10.1016/j.cub.2005.06.061)
2. Almany GR, Berumen ML, Thorrold SR, Planes S, Jones GP. 2007. Local replenishment of coral reef fish populations in a marine reserve. *Science* **316**, 742-744. (doi:10.1126/science.1140597)
3. Planes S, Jones GP, Thorrold SR. 2009. Larval dispersal connects fish populations in a network of marine protected areas. *Proc. Natl. Acad. Sci. USA* **106**, 5693–5697. (doi: 10.1073/pnas.0808007106)
4. Saenz-Agudelo P, Jones GP, Thorrold SR, Planes S. 2011. Connectivity dominates larval replenishment in a coastal reef fish metapopulation. *Proc. R. Soc. B*. **278**, 2954-2961. (doi:10.1098/rspb.2010.2780)
5. Beldade R, Holbrook SJ, Schmitt RJ, Malone D, Bernardi G. 2012. Larger female fish contribute disproportionately more to self-replenishment. *Proc. Biol. Sci. B*. **279**, 2116-2121. (doi:10.1098/rspb.2011.2433)
6. Berumen ML, Almany GR, Planes S, Jones GP, Saenz-Agudelo P, Thorrold SR. 2012. Persistence of self-recruitment and patterns of larval connectivity in a marine protected area network. *Ecol. Evol.* **2**, 444-452. (doi: 10.1002/ece3.208)
7. Saenz-Agudelo P, Jones GP, Thorrold SR, Planes S. 2012. Patterns and persistence of larval retention and connectivity in a marine fish metapopulation. *Mol. Ecol.* **21**, 4695-4705. (doi: 10.1111/j.1365-294X.2012.05726.x)
8. D’Aloia CC, Bogdanowicz SM, Majoris JE, Harrison RG, Buston PM. 2013. Self-recruitment in a Caribbean reef fish: a new method for approximating dispersal kernels accounting for seascape. *Mol. Ecol.* **22**, 2563-2572. (doi:10.1111/mec.12274)
9. Madduppa HH, Timm J, Kochzius M. 2014. Interspecific, spatial and temporal variability of self-recruitment in anemonefishes. *PLoS ONE* 9:e90648.
10. Schunter C, Pascual M, Garza JC, Raventos N, Macpherson E. 2014. Kinship analyses identify fish dispersal events on a temperate coastline. *Proc. R. Soc. B*. **281**, 20140556. (doi:10.1098/rspb.2014.0556)
11. Nanninga G, Saenz-Agudelo P, Zhan P, Hoteit I, Berumen ML. 2015. Not finding Nemo: limited reef-scale retention in a coral reef fish. *Coral Reefs* **34**, 383-392. (doi:10.1007/s00338-015-1266-2)
12. Salles OC, Maynard JA, Joannides M, Barbu CM, Saenz-Agudelo P, Almany GR, Berumen ML, Thorrold SR, Jones GP, Planes S. 2015. Coral reef fish populations can persist without immigration. *Proc. R. Soc. B*. **282**, 20151311. (doi:10.1098/rspb.2015.1311)
13. Jones GP, Milicich MJ, Emslie MJ, Lunow CJ. 1999. Self-recruitment in a coral reef fish population. *Nature* **402**, 802–804. (doi:10.1038/45538)
14. Swearer SE, Caselle JE, Lea DW, Warner RR. 1999. Larval retention and recruitment in an island population of a coral-reef fish. *Nature* **402**, 799-802. (doi:10.1038/45533)
15. James MK, Armsworth PR, Mason LB, Bode L. 2002. The structure of reef fish metapopulations: modelling larval dispersal and retention patterns. *Proc. R. Soc. Lond. B* **269**, 2079–2086 (doi:10.1098/rspb.2002.2128)
16. Patterson HM, Kingsford MJ, McCulloch MT. 2005. Resolution of the early life history of a reef fish using otolith chemistry. *Coral Reefs* **24**, 222–229. (doi:10.1007/s00338-004-0469-8)
17. Patterson HM, Swearer SE. 2007. Long-distance dispersal and local retention of larvae as mechanisms of recruitment in an island population of a coral reef fish. *Austral. Ecol.* **32**, 122-130. (doi:10.11111/j.1442-9993.2006.01669.x)
18. Hamilton SL, Regetz J, Warner RR. 2008. Post-settlement survival linked to larval life in a marine fish. *Proc. Natl. Acad. Sci. USA* **105**, 1561–1566. (doi: 10.1073/pnas.0707676105)
19. Harrison HB, Williamson DH, Evans RD, Almany GR, Thorrold SR, Russ DR, Feldheim KA, van Herwerden L, Planes S, Srinivasan M, Berumen ML, Jones GP. 2012. Larval export from marine reserves and the recruitment benefit for fish and fisheries. *Curr. Biol.* **22**, 1023–1028. (doi:10.1016/j.cub.2012.04.008)
20. Hogan JD, Thiessen RJ, Slae PF, Heath DD. 2012. Local retention, dispersal and fluctuating connectivity among populations of coral reef fish. *Oecologia* **168**, 61-71. (doi:10.1007/s00442-011-2058-1)
21. Almany GR, Hamilton RJ, Bode M, Matawi M, Potuku T, Saenz-Agudelo P, Planes S, Berumen ML, Rhodes KL, Thorrold SR, Russ GR, Jones GP. 2013. Dispersal of grouper larvae drives local resource sharing in a coral reef fishery. *Curr. Biol.* **23**, 626-630. (doi: 10.1016/j.cub.2013.03.006)
22. Chittaro PM, Hogan JD. Patterns of connectivity among populations of a coral reef fish. *Coral Reefs* **32**, 341-354. (doi:10.1007/s00338-012-0990-0)
23. Al-Salamah, M. Connectivity of the long fin grouper (*Ephinephelus quoyanus*) in a marine reserve in the Great Keppel Island Group. 2014.
24. Lozano-Cortes D. Self-recruitment in the bumphead parrotfish under different levels of fishing pressure in the Solomon Islands. 2014.
25. Cuif M, Kaplan DM, Fauvelot C, Lett C, Vigliola L. 2015. Monthly variability of self-recruitment for a coral reef damselfish. *Coral Reefs* **34**, 759-770. (doi:10.1007/s00338-015-1300-4)
26. Sapolu T. Age-based demography and reproductive ontogeny of angelfishes belonging to the family Pomacantidae. 2005.
27. Srinivasan M, Jones GP. 2006. Extended breeding and recruitment period of fishes on a low latitude coral reef. *Coral Reefs* **25**, 673-682. (doi:10.1007/s00338-006-0153-2)
28. Raymond M, Rousset F. 1995. GENEPOP: population genetics software for exact tests and ecumenicism. *J. Hered.* **86**, 248-249.
29. Benjamini Y, Hochberg Y. 1995. Controlling the false discovery rate: a practical and powerful approach to multiple testing. *J. R. Stat. Soc. Series B (Methodol.)* **57**, 289-300.
30. Jones O, Wang J. 2010. COLONY: a program for parentage and sibship inference from multilocus genotype data. Mol. Ecol. Res. **10**, 551–555. (doi:10.1111/j.1755-0998.2009.02787.x)
31. Gerber S, Chabrier P, Kremer A. 2003. FAMOZ: a software for parentage analysis using dominant, codominant and uniparentally inherited markers. *Mol. Ecol. Notes.* **3**, 479–481. (doi:10.1046/j.1471-8286.2003.00439.x)
32. Pritchard JK, Stephens M, Donelly P. 2000. Inference of population structure using multilocus genotype data. *Genetics* **155**, 945–959.
33. Earl DA, von Holdt BM. 2012. STRUCTURE HARVESTER: A website and program for visualizing STRUCTURE output and implementing the Evanno method. *Conserv. Genet. Resour.*. **4**, 359-361. (doi:10.1007/s12686-011-9548-7)
34. Kopelman NM, Mayzel J, Jakobsson MJ, Rosenberg NA, Mayrose I. 2015. CLUMPAK: a program for identifying clustering modes and packaging population structure inferences across K. Mol. Ecol. Res. **15**, 1179–1191. (doi:10.1111/1755-0998.12387)
35. Peakall R, Smouse PE. 2012. GENALEX 6.5: genetic analysis in Excel. Population genetic software for teaching and research-an update. *Bioinformatics* **28**, 2537-2539. (doi:10.1093/bioinformatics/bts460)
